# Associations between Extending Access to Primary Care and Emergency Department Visits: A Difference-In-Differences Analysis

**DOI:** 10.1371/journal.pmed.1002113

**Published:** 2016-09-06

**Authors:** William Whittaker, Laura Anselmi, Søren Rud Kristensen, Yiu-Shing Lau, Simon Bailey, Peter Bower, Katherine Checkland, Rebecca Elvey, Katy Rothwell, Jonathan Stokes, Damian Hodgson

**Affiliations:** 1 Manchester Centre for Health Economics, University of Manchester, Manchester, United Kingdom; 2 Alliance Manchester Business School, University of Manchester, Manchester, United Kingdom; 3 NIHR School for Primary Care Research Manchester Academic Health Science Centre, University of Manchester, Manchester, United Kingdom; 4 National Institute for Health Research Collaboration for Leadership in Applied Health Research and Care Greater Manchester, University of Manchester, Manchester, United Kingdom; 5 National Institute for Health Research Collaboration for Leadership in Applied Health Research and Care Greater Manchester, Salford Royal NHS Foundation Trust, Salford, United Kingdom; 6 NIHR Greater Manchester Primary Care Patient Safety Translational Research Centre, Manchester Academic Health Science Centre, University of Manchester, Manchester, United Kingdom; Stanford University, UNITED STATES

## Abstract

**Background:**

Health services across the world increasingly face pressures on the use of expensive hospital services. Better organisation and delivery of primary care has the potential to manage demand and reduce costs for hospital services, but routine primary care services are not open during evenings and weekends.

Extended access (evening and weekend opening) is hypothesized to reduce pressure on hospital services from emergency department visits. However, the existing evidence-base is weak, largely focused on emergency out-of-hours services, and analysed using a before-and after-methodology without effective comparators.

**Methods and Findings:**

Throughout 2014, 56 primary care practices (346,024 patients) in Greater Manchester, England, offered 7-day extended access, compared with 469 primary care practices (2,596,330 patients) providing routine access. Extended access included evening and weekend opening and served both urgent and routine appointments. To assess the effects of extended primary care access on hospital services, we apply a difference-in-differences analysis using hospital administrative data from 2011 to 2014. Propensity score matching techniques were used to match practices without extended access to practices with extended access. Differences in the change in “minor” patient-initiated emergency department visits per 1,000 population were compared between practices with and without extended access.

Populations registered to primary care practices with extended access demonstrated a 26.4% relative reduction (compared to practices without extended access) in patient-initiated emergency department visits for “minor” problems (95% CI -38.6% to -14.2%, absolute difference: -10,933 per year, 95% CI -15,995 to -5,866), and a 26.6% (95% CI -39.2% to -14.1%) relative reduction in costs of patient-initiated visits to emergency departments for minor problems (absolute difference: -£767,976, -£1,130,767 to -£405,184). There was an insignificant relative reduction of 3.1% in total emergency department visits (95% CI -6.4% to 0.2%). Our results were robust to several sensitivity checks. A lack of detailed cost reporting of the running costs of extended access and an inability to capture health outcomes and other health service impacts constrain the study from assessing the full cost-effectiveness of extended access to primary care.

**Conclusions:**

The study found that extending access was associated with a reduction in emergency department visits in the first 12 months. The results of the research have already informed the decision by National Health Service England to extend primary care access across Greater Manchester from 2016. However, further evidence is needed to understand whether extending primary care access is cost-effective and sustainable.

## Abstract 2

William Whittaker and colleagues use difference-in-differences analysis to determine whether extending access to primary care is linked with fewer emergency department visits.

## Author Summary

**Why Was This Study Done?:**

- Recent policy in England has focused on providing a 7-day National Health Service, which includes extending access to primary care outside of normal working hours (in the evenings and on weekends).
- There is limited evidence that improving access to primary care by providing additional appointments outside of routine working hours reduces demand at emergency departments.
- National Health Service Greater Manchester provided £3.1 million to enable several groups of practices to provide additional non-working hour appointments during 2014; this study evaluates the effects of these appointments on emergency department attendances.

**What Did the Researchers Do and Find?:**

- The change in emergency department attendance in 2014 compared to average attendance in 2011–2013 was calculated for 56 practices (346,024 patients) with additional non-working hour appointments and compared to 469 practices (2,596,330 patients) without additional appointments in Greater Manchester.
- In 2014, emergency department attendance for patients self-referring with minor conditions dropped by 26.4% (10,933 fewer visits) in comparison to practices without additional appointments.
- Emergency department costs for treating patients self-referring with minor conditions reduced by £767,976 in comparison to practices without additional appointments.

**What Do These Findings Mean?:**

- This study provides evidence showing additional primary care appointments outside of working hours may reduce attendance at emergency departments but may not be cost-saving to the health service as a whole.
- The findings of the study may not be observed in areas unlike Greater Manchester, England.
- Practices could opt in to providing additional appointments. Whilst we compare similar practices on the characteristics we can observe, unobservable differences may restrict the generalisability of the findings of the study.

## Introduction

In common with health services worldwide, the United Kingdom National Health Service (NHS) faces high demand and cost pressures. In 2014–15, there were 18.5 million visits at emergency departments in hospitals in England, 20.9% of which resulted in a hospital admission [1]. The annual cost of non-admitted emergency department activity in 2013–14 was £2,300 million (US$3,500/€3,200 million), representing 4% of total hospital costs and 2.2% of total health expenditure [2].

High-quality primary care services improve health and reduce mortality and health inequalities [3]. In the UK NHS, accessible, integrated health care services, with primary care at their core, feature across the political spectrum [4]. Financial pressures on the NHS in England have led to a policy focus on improving access to primary care, both to deliver a more convenient service for patients and, in doing so, to relieve pressures on hospital care. To this end, the UK government has invested £150 million (US$229/€207 million) since 2013 in a number of regional initiatives to extend access to primary care across England, as part of a long-term plan to extend seven-day working across the entire NHS.

A factor which may limit the impact of primary care on use of emergency departments is opening hours of primary care facilities. In the UK, primary care services are provided by general practitioners (GPs), and conventional working hours are approximately 8:30 a.m. to 6:30 p.m., Monday to Friday, with out-of-hours services provided by a contracted provider. In England, 26.5% of unplanned emergency department use followed unsuccessful attempts to access primary care [5], and use of other types of urgent care also arise out of similar issues [6,7]. Extending access to primary care beyond conventional hours (so-called “out-of-hours” or “after hours”‘ services) may reduce emergency department use by providing patients with an alternative to visiting the emergency department.

Studies evaluating the impact of extended out-of-hours services on emergency department visits are sparse. In a systematic review, Ismail et al. (2013) found limited and mixed evidence: studies used before-after analysis and, hence, lacked a comparator, and focus has been on out-of-hours urgent primary care visits rather than pre-booked appointments [8]. In several observational studies in England, patients with better access to primary care (typically defined in the studies as the availability of an appointment within 48 h) have been found to have lower hospital admissions, though these studies have been condition-specific, restricting generalisability to the general population [9–14]. Cowling et al. (2015) highlight the need for more robust analyses on the effects of improving access to primary care on the use of other services, outcomes, and costs [4].

We investigated the association between extending opening hours in primary care and emergency department visits for minor conditions, using hospital administrative data to compare populations in practices that did and did not extend open hours.

## Methods

Ethical approval was not required for this study since the study was an evaluation of routine emergency department service use with non-randomisation in process or to allocation to treatment. Data received for the analyses were de-identified.

In the UK, primary care practices usually provide routine and urgent (same-day) appointments between the hours of 8:30 a.m. and 6:30 p.m., Monday to Friday. Outside these times, patients with urgent problems can access “out-of-hours” services provided outside of general practice, which include urgent assessment by a doctor if required.

In 2014, NHS England (part of the UK Department of Health, which plans and oversees delivery of the English NHS) provided funding for a number of sites that would deliver innovations in integrated care, use of technology, and extended access to primary care. Invitations to bid for funding were sent to all practices in Greater Manchester. Eighteen schemes were submitted, each varied in the number of practices involved as part of the scheme and with the involvement of primary care organisations (Clinical Commissioning Groups [CCGs] responsible for commissioning health care services for their local population of around 200–300,000 people). The formation of practices within schemes was self-determined by practices, but in some instances was determined by the CCG. A panel including the Chief Executive of NHS England Greater Manchester, a senior commissioner, an integrated care lead, and senior medical and nursing staff reviewed each scheme.

Six schemes were successful in obtaining funding totalling £4.1 million (US$6.3/€5.6 million). Our analysis concerns four schemes that focused on extending opening hours in primary care, the four schemes received total funding of £3.1 million (US$4.7/€4.3 million). These schemes offered a combination of additional urgent and routine GP appointments of between 10 and 15 min, in the evenings Monday to Friday (approximately 5 p.m. to 9 p.m.) and on both days of the weekend (see Table 1 for specific details). The additional appointments were accessible both to patients requesting an urgent appointment and to those for whom booking an evening or weekend appointment was more convenient.

**Table 1:** Intervention description: Enhanced access to “out-of-hours” primary care in each intervention practice Clinical Commissioning Group*.

	Coverage and funding	Hours of operation and staffing
**Bury CCG**	6/35 practices (c.32,894). £765,000 (US$1,169,341/€1,053,329)	6:30–8 p.m. Monday–Friday, 8 a.m.–6 p.m. Saturday and Sunday. 2 GPs and receptionists. 18 x 10 min appointments per day Monday–Friday, 120 x 10 min appointments per day Saturday and Sunday.
**Central Manchester CCG**	33/35 practices (c.203,982). £979,000 (US$1,496,450/€1,053,329)	6–8 p.m. Monday–Friday, 9–11 a.m. Saturday and Sunday. 1 GP and 2 receptionists. 12 x 10 min appointments per day, Monday–Sunday.
**Heywood, Middleton, Rochdale CCG (Heywood scheme)**	6/39 practices (c.30,890). £630,000 (US$962,987/€867,447)	4–9 p.m. Monday–Friday, 9:30 a.m.–9 p.m. Saturday and 1:30 p.m.—9 p.m. Sunday. 2 GPs after 6 wk. 28 x 15 min appointments per day Monday–Friday 51 x 15 min appointments per day Saturday and 34 x 15 min appointments per day Sunday.
**Heywood, Middleton, Rochdale CCG (Middleton scheme)**	8/39 practices (c.51,680). £770,000 (US$1,176,984/€1,060,213)	6:30–9:30 p.m. Monday–Friday, 6–9 p.m. Saturday and Sunday. 1 GP 18 x 10 min appointments per day Monday–Sunday.

*Clinical Commissioning Groups are responsible for commissioning health care services for their local population of around 200–300,000 people.

**Fig 1:**
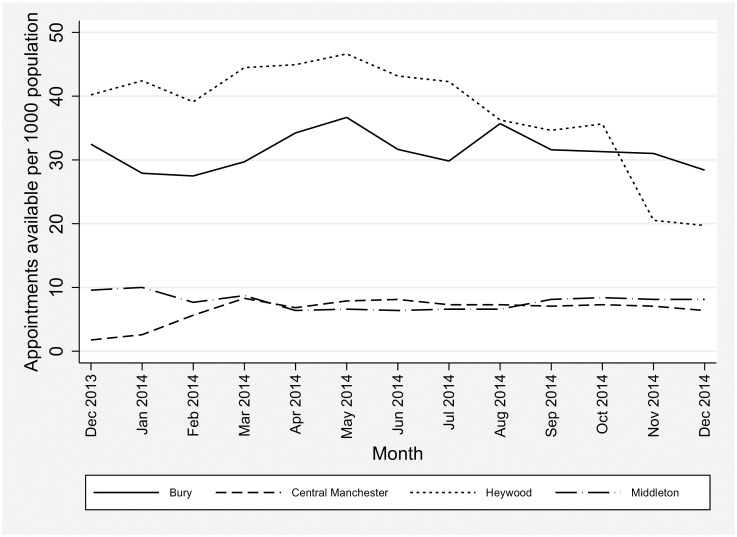
Additional appointments available per 1,000 patients.

The schemes varied considerably in cost and in scale; the largest served a population of over 200,000—almost four times the size of the next largest. An average of approximately 35 additional hours of appointments were available per scheme per week (ranging from 14–60 h per week). The schemes also varied in their workforce arrangements, with two using a combination of local GPs and locums, and two contracting with the local “out-of-hours” provider. All four schemes received funding for extended access from December 2013. In total there were 10.25 additional appointments per 1,000 registered patients available in January 2014; the number of additional appointments remained fairly constant over the year with 10.22 additional appointments per 1,000 patients available in December 2014 (Fig 1).

We used existing hospital administrative data for analysis. This data has complete population coverage for all emergency department visits. Walk-in centre activity was excluded since the data is incomplete for this activity. Data are coded as to type of referral (e.g., patient-initiated, provider-initiated) and problem intensity (“minor,” “standard,” “high,” see Table 2 for details). All statistical analysis was carried out using the statistical package Stata (version 13). Our analysis uses data for Greater Manchester, which has the benefit of enabling comparisons to practices within the same environment and with similar practice populations.

**Table 2:** Emergency department outcomes.

Emergency department use	Description
**Patient-initiated (minor intensity)**	Patient-initiated emergency department use for minor-intensity problems
**Cost Patient-initiated (minor intensity)**	Cost of Patient-initiated use for minor-intensity problems
**Total**	Total emergency department use
**Intensity type** ^	
**Minor**	Emergency department use with intensity type coded as HRG V07 or V08 (2010/11) or VB10Z, VB11Z, VB06Z, or VB09Z in subsequent years
**Standard**	Emergency department use with intensity type coded HRG V05 or V06 (2010/11) or VB07Z or VB08Z in subsequent years
**High**	Emergency department use with intensity type coded with HRG V01,V02,V03 orV04 (2010/11) or VB01Z, VB02Z, VB03Z, VB04Z or VB05Z in subsequent years.
**Intensity missing**	Emergency department use with missing data on intensity type code with HRG code DOA, N/A, U06, UZ01Z
**Referral type**	
**GP-referral**	GP referral for emergency department use
**Patient-initiated**	Patient-initiated Emergency department use
**Other referral**	Emergency department use with other mode of referral
**Code missing**	Emergency department use with missing data on mode of referral

HRG: Health care Resource Group

HRG codes group clinically similar treatments on the basis of resource used.

^The grouping of HRG codes follows the same minor/standard/high grouping as the 2010/11 HRG tariff.

Our principal analysis concerns patient-initiated visits to emergency departments for “minor” problems. This measure was chosen as increasing primary care open hours is most likely to impact on problems that could be adequately managed in primary care. We conduct secondary analysis for total emergency department visits and also report data on other referral types (e.g., referrals from primary care) to assess whether the intervention affects referral patterns and “standard” and “high” intensity visits.

The proposal contains a range of proposed analyses (S1 Text). Unlike a standard trial with accompanying protocol, the evaluation needed to be responsive to what was an exploratory and time-limited pilot scheme. The bids that were initially submitted by each scheme contained many services to be piloted and many outcomes that were the proposed target of these services. This included not only extending the hours that primary care services could be made available but also extending the range of services on offer; for example, extended diagnostic services or specialist outreach clinics for long-term conditions. Within the first few months of the funding, each scheme narrowed its focus substantially, so that in the four schemes in question, establishing an extended hours service became the main focus and plans for extending the range of services were dropped. This then meant that the outcomes that might have been associated with these services (including hospital admissions and emergency department use for more severe health conditions) were also dropped. This meant the only feasible impact the pilot schemes could have on secondary care was minor attendance.

In total we have data on emergency department visits covering 2,942,354 patients from 525 primary care practices in Greater Manchester from 2011_2014. Fifty-six practices (10.7%, with 346,024 patients) participated in one of the four schemes offering extended opening hours during the intervention period. Secondary Uses Service (SUS) Payment by Results data for Accident and Emergency services were provided by the North West Commissioning Support Unit through NHS England. The data were provided at the patient level and aggregated to primary care practices, coded as either intervention practices (offering extended opening hours) or comparator practices (offering routine opening hours and excluding practices involved in one of the two funded schemes with no additional appointments). The data do not allow us to assess whether patients visited primary care before attending the emergency department unless they were referred to the emergency department by the primary care practitioner (“GP-referral”). Emergency department use was measured quarterly per 1,000 registered practice population. We assessed the effects of the intervention on costs of emergency department services by applying standard Payment by Results tariffs for financial year 2013/14 for each type of use. A single (2013/14) tariff was applied to all periods to ensure variations in costed activity over the period reflected activity changes rather than unit cost fluctuations.

### Principal Analysis

Relative differences in emergency department visits per 1,000 registered population between intervention practice and comparators before (2011–2013) and after (2014) the intervention were estimated using regression analysis of *difference-in-differences* [15]. We tested for statistically significant differences in emergency department use between intervention and comparator using an interaction term for intervention with a binary indicator for the intervention period. The interaction term gives the relative (“risk”) difference in emergency department use for the intervention practices (the change in use over time beyond the change observed in the comparison group—the “difference in differences”).

Estimation was made using Ordinary Least Squares regressions with 525 practice level binary indicators (practice fixed-effects) and 15 time quarter indicators (time fixed-effects). The practice fixed-effects control for any time-invariant confounding in emergency department use between practices that may be associated with the intervention (for example, if intervention practices have higher or lower number of emergency department visits generally). Time fixed-effects control for any temporal fluctuations (such as seasonal effects) in emergency department use from the general trend. Standard errors were corrected for heteroscedasticity to account for potential heterogeneity in the error term (which can result in the variance of estimates being biased and hence biases statistical inference).

Absolute risk/difference in 2014 was obtained by applying the estimated percentage point reduction to the average rate of attendances per 1,000 and multiplied by four (quarters) and the population size (divided by 1,000).

### Confounding

To be unbiased, the difference-in-differences approach makes several assumptions [16,17]. First, there should be no confounding due to selection into intervention. Second, both intervention and comparator practices should demonstrate similar trends in emergency department use over time prior to the intervention.

Confounding due to selection is a concern as primary care practices may self-select into the intervention. The use of primary care practice fixed-effects removes some of the potential confounding due to self-selection; namely, the fixed-effects approach accounts for time-invariant characteristics of practices that may account for historic differences in the outcome between intervention and comparators. Residual confounding may still occur should historic differences not fully identify whether the patients or practitioners in intervention practices are more or less likely to embrace the intervention than patients or practitioners in comparator practices.

To account for potential selection into the intervention group and minimize the derived bias in the estimated impact, we match intervention practices to comparator practices via propensity score matching so that, conditional on observable differences between comparator and intervention groups, the propensity of treatment is similar for both groups. Potential outcomes under these matched groups are then independent of treatment assignment conditional on the observable differences [18]. This conditional independence of treatment assignment is useful in observational studies in which treatment allocation is non-random and can be viewed as an approach that seeks to replicate random assignment in conventional randomised controlled trials.

We estimated the propensity to be in the intervention group via a probit model of intervention group membership against observed practice covariates hypothesised to relate to intervention assignment and the outcome (emergency department use) but not affected themselves by the intervention. The covariates used include practice practitioner characteristics (age, gender, country of qualification, and the size of registered patients per practitioner) and practice patient characteristics (age, gender, deprivation, and limited long-standing illness). Data on practice practitioner characteristics were derived from data from the Health and Social Care Information Centre on registered practitioners [19] and data on patient characteristics from the General Practice Patient Survey, a national sample survey used with all English primary care practices, and weighted to produce representative samples of practice populations [20]. The practice, practitioner, and patient characteristics may influence the relative volume and type of emergency hospital use, and may result in different responses to the intervention of extended access. On the one hand, practitioner’s motivation to provide extended appointments may vary with the gender, age, and ethnicity of practitioners and may be influenced by the current strains on appointments and the culture within the practice. On the other hand, the demand and subsequent uptake of extended access appointments may be hypothesised to be correlated with the age, gender, deprivation, and need structure of a practice’s patient base.

We excluded practices outside the common support (overlapping propensity scores) from both intervention and comparator groups. We constructed our matched comparator and intervention groups under a variety of matching strategies: a) nearest neighbour with replacement (whereby each intervention practice is matched to *n* comparator practices based on propensity score closest proximity) [18]; b) radius matching (whereby each intervention practice is matched to all comparator practices where the propensity score falls within a given radius) [21]; c) kernel weighting (whereby all comparator practices within a bandwidth are matched to the intervention practice but weighted in relation to the distance in propensity score) [22]; d) all comparator practices under the common support range (whereby all comparator practices have equal weight but must fall under the common support); and e) the top 25% of comparator practices under common support (whereby only those comparator practices in the top quartile of ranked propensity score are matched to the intervention practices) [23]. Among the matched comparator and intervention groups identified with each strategy, we selected the one that best meets the acceptability criteria of covariate balance. Covariate balance was assessed via standardised differences (bias) and variance ratios. All propensity score matching was conducted using psmatch2 in Stata [24].

Confounding may also arise should either the intervention or comparator practices have existing access outside of core hours. We are unable to identify the extent of existing access outside of core hours over the period studied, but as the scheme extended existing hours of primary care access, our results can be considered as a conservative estimate of the impact extended access has on emergency department use.

The assumption of similarity of trends was tested on the matched sample by estimating a linear time trend interacted with the intervention dummy in the pre-intervention period. This was performed separately for each type of emergency department use modelled. Where divergent time trends are observed, the difference-in-differences analysis may be biased since the estimated difference may be reflective of differences in trends of the outcome variable.

All variables were transformed using the inverse hyperbolic sine transformation which can be interpreted roughly as percentage changes. This is similar to the logarithmic transformation but can be used when the dependent variable takes zero values [25].

### Sensitivity Analyses

We conducted additional analyses to assess the robustness of our results:

1. Total attendance: We estimated effects on total emergency department use, excluding those patients eventually admitted to hospital (since these could be judged as “appropriate” use of emergency department).
2. 6-mo intervals: We estimated effects at 6-mo intervals to see if the effects of the intervention were more apparent in later time periods after introduction of the intervention.
3. Regression to the mean: To assess whether our results are subject to bias caused by regression to the mean (representing random fluctuations around a long-run average), we replicate our models with baseline emergency department use included; as this is time invariant this removes the potential to include practice fixed-effects.
4. Equal pre- and post-intervention comparisons: We estimate the model using only 2013 quarters as the pre-intervention period. This approach compares pre- and post-intervention periods close to the intervention start; in the current context this could be 1 y pre- (2013) and 1 y post-intervention (2014).
5. Model specification: The log-linear approach taken assumes a normal distribution in the error term, which is unlikely to hold under count data. We estimate negative binomial models to see whether our results were robust to model specification.
6. Unmatched sample with time-trend adjustment: As an alternative to matching, we estimated effects on the unmatched sample including all practices in Greater Manchester. Where the difference in trends between intervention and comparators was statistically significant, we predicted emergency department use by extrapolating the time trend in the pre-intervention period to the intervention period. Time-trend adjusted emergency department use was then calculated as the difference between actual and predicted use at each time point and used as the outcome of interest. This approach controls for the part of the estimated effect of the intervention that is due to differences in trends before the intervention under the assumption that practices would have continued along their pre-intervention trends, and has been used in previous health research [26,27].

A number of changes to the analysis plan were made during the peer review process, including the addition of propensity score matching to address imbalance and reduce the potential for divergent time trends; the inclusion of practice characteristics in comparisons of matches between practices; and suggestions for sensitivity analysis regarding equal pre- and post-periods and investigations of modelling negative binomial models as opposed to linear models.

## Results

**Fig 2:**
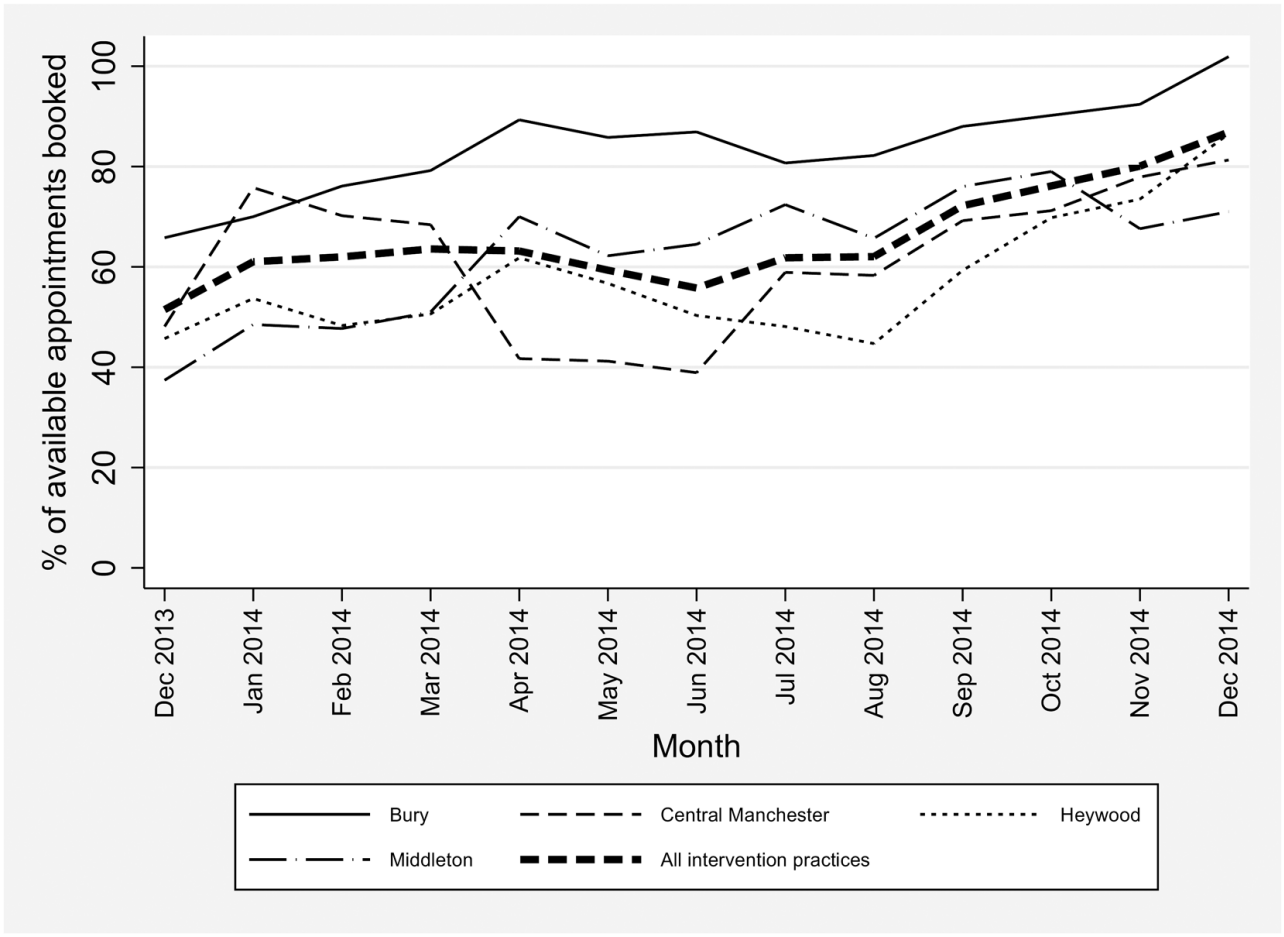
Percentage of additional appointments booked in intervention practices, by Clinical Commissioning Group.

There were 51,465 additional appointments provided by the intervention during the extended opening hours (152 appointments per 1,000 population). Numbers of additional appointments varied between the intervention practices, from 10/1,000/month to 40/1,000/month. In total, 65.1% (33,519/51,465) of additional appointments were used, with an increase over time (see Fig 2), perhaps reflecting the gradual embedding of the service and increased patient awareness.

### Matching

Assessing the quality of the matched samples requires consideration of the similarity across the comparator and intervention (treated) group in both the standardised differences and variance of the observed covariates and propensity scores. Student’s *t* tests of equivalent covariates are uninformative due to issues of reduced sample size; a joint test of the significance of all observed covariates may highlight imbalance but is also suspect to this reduced sample concern. Austin (2009) recommends that comparisons between unmatched and matched samples be made on the basis of standardised differences and relative variances of both the covariates and propensity score since these are not sensitive to sample size [28]. This ensures the comparator and intervention groups are similar both in the mean of the covariates and the distribution of these covariates in addition to the overall propensity score. Equivalent control and intervention groups would have similar means (a low percentage bias) and ratios of variances close to unity. Rubin (2001) provides recommendations on the diagnostic criteria of the propensity score, recommending that the absolute standardised difference of the means of the linear index of the propensity score between the comparator and intervention (treated) group is below 25% [29]. Rubin (2001) also recommends that the variance ratio of the propensity score lies between 0.5 and 2 at the extremes.

Diagnostic results for a range of matching procedures are reported in S1 Table. All matching was conducted under common support of the propensity score; one comparator practice lay outside the range of common support. Although balance in the observed covariates and acceptable variance ratios were generally met across each procedure, the acceptable percentage bias of less than 25% was only met by the kernel matching procedure using the epanechnikov kernel and bandwidth 0.06. Our analysis was therefore conducted on the kernel matched sample. The percentage bias for each covariate under the unmatched and matched sample using kernel matching are reported in S2 Table. The percentage bias in the matched and unmatched samples are plotted in S1 Fig.

Kernel matching generates weights for comparator practices that are determined by the distance in propensity scores to intervention practices. We weight the difference-in-differences models using the kernel weights generated by the propensity score model. Since the weights used in the regression models are based on propensity scores (predicted values) there are concerns over the validity of the standard errors [30]. Abadie and Imbens (2008) note that unlike nearest-neighbour matching (which matches a finite, restricted number of comparators to intervention practices), the number of matches increase with sample size under kernel matching; under these circumstances bootstrapping is likely to generate valid inference [31]. Standard errors in each model were obtained using bootstrapping with 1,000 replications simultaneously over both the propensity model and the difference-in-differences model.

### Principal analysis

**Fig 3:**
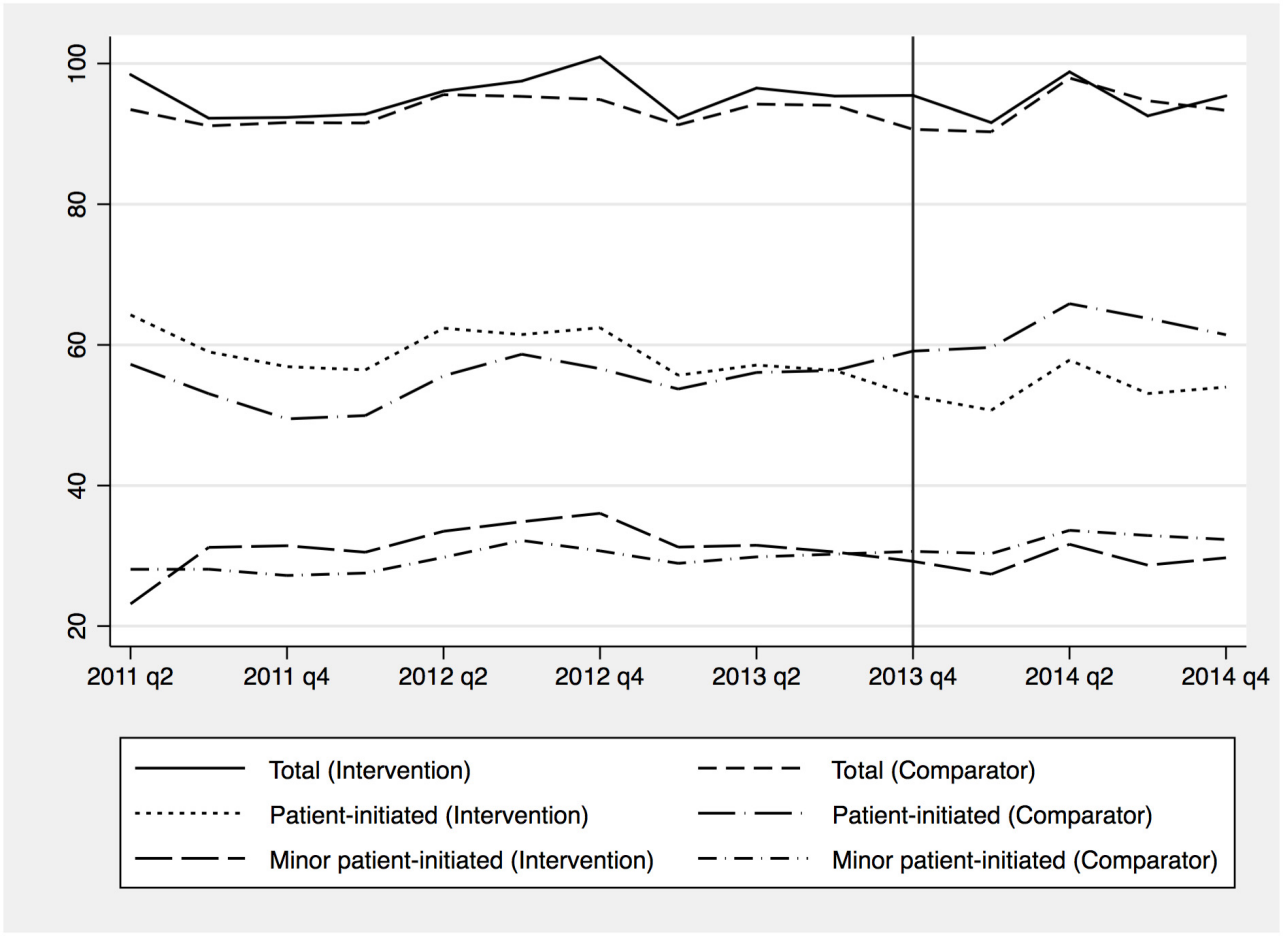
Average emergency department use per 1,000 registered patients per quarter by year.

Table 3 and Fig 3 report the pre- and post-period emergency department use for each outcome measure. On average, there were approximately 95 attendances at emergency departments per 1,000 per quarter, with approximately half being for “minor” problems, and patient-initiated use making up the largest proportion.

**Table 3:** Average emergency department use per 1,000 registered patients in the pre- (2011 to 2013) and post- (2014) intervention period and difference-in-differences estimates of changes in emergency department use.

		Average attendance		Estimated difference in 2011–2013 trend*	Difference-in-differences estimate**		
		C	I	Estimate [95% Confidence Interval]	Estimate	95% Confidence interval	*p*-value
**Patient-initiated referrals (minor intensity)**	Pre	29.4	31.2				
	Post	32.3	29.4	-0.004 [-0.015 to 0.007]	-26.39%	[-38.61% to -14.16%]	(<0.001)
**Cost of patient-initiated referrals (minor intensity)**	Pre	2,007.3	2,171.9				
	Post	2,218.4	2,061.0	-0.008 [-0.018 to 0.002]	-26.63%	[-39.21% to -14.05%]	(<0.001)
**Total**	Pre	93.1	95.4				
	Post	94.1	94.6	0.002 [-0.002 to 0.006]	-3.08%	[-6.39% to 0.24%]	(0.069)
**Intensity type**							
**Minor**	Pre	46.8	48.2				
	Post	46.8	47.5	0.007 [-0.003 to 0.016]	-4.45%	[-9.18% to 0.28%]	(0.065)
**Standard**	Pre	35.3	39.1				
	Post	36.1	38.4	-0.005 [-0.010 to -0.001]	-5.42%^	[-9.86% to -0.90%]	(0.019)
**High**	Pre	8.5	6.4				
	Post	10.7	8.5	-0.002 [-0.017 to 0.014]	-1.08%	[-5.52% to 7.96%]	(0.722)
**Missing**	Pre	2.5	1.8				
	Post	0.4	0.1	0.016 [-0.022 to 0.054]	11.31%	[-0.54% to 22.14%]	(0.040)
**Referral type**							
**GP-referral**	Pre	3.6	2.0				
	Post	3.9	2.5	0.002 [-0.010 to 0.013]	4.43%	[-4.11% to 12.74%]	(0.315)
**Patient-initiated**	Pre	55.1	58.6				
	Post	62.7	53.9	-0.022 [-0.031 to -0.013]	-31.88%^	[-44.80% to -18.91%]	(<0.001)
**Other referral**	Pre	33.9	32.9				
	Post	27.4	38.1	0.037 [0.025 to 0.049]	33.79%^	[21.37% to 46.22%]	(<0.001)
**Code missing**	Pre	0.4	1.9				
	Post	0.1	0.1	-0.136 [-0.172 to -0.099]	-38.27%^	[-48.65% to -27.76%]	(<0.001)

C = comparator group; I = intervention group

Intervention group is matched Greater Manchester intervention practices, and comparator group is all Greater Manchester matched non-intervention practices; sample size for each model is 7,304; this is the matched (weighted) sample using kernel propensity score matching.

Bootstrapped standard errors (1,000 replications) over both propensity score and regression models.

*Estimated difference in trend is estimated from an Ordinary Least Squares regression of attendance regressed on a linear time trend and an intervention practice interacted linear time trend; the estimate provided gives the estimated divergence of the intervention practices time trend in comparison to the comparator practices time trend.

**All activities were transformed using the inverse hyperbolic sine transformation; estimate gives the relative (risk) difference in emergency department use for intervention versus comparators; each estimate is obtained from a separate difference-in-differences Ordinary Least Squares regression.

^ Divergent time trends—the difference-in-differences assumption of equivalent time trends is not satisfied and inference should not be made on these estimates.

Table 3 provides average pre- and post-intervention observed attendance, an estimate of the difference in time trends between matched comparator and intervention practices in the pre-intervention period, and the difference-in-differences estimates. Statistically significant differences in pre-intervention trends between comparator and intervention practices were found for standard intensity, patient-initiated, other, and missing referrals attendance. For these, the difference-in-differences estimate is biased and inference should not be made on these estimated effects (denoted by ^ in Table 3). Intervention practices demonstrated a 26.4% relative reduction in patient-initiated referrals to emergency departments for “minor” problems (95% confidence interval [CI] -38.6% to -14.2%; absolute risk/difference: -10,933 per year, 95% CI -15,995 to -5,866), which in turn led to a 26.6% relative reduction in the costs of patient-initiated referrals for “minor” problems (95% CI -39.2% to -14.1%; absolute difference: -£767,976, 95% CI -£1,130,767 to -£405,184) (-US$1,173,890 [95% CI -US$1,728,434, to -US$619,344]; -€1,057,426 [95% CI -€1,556,953 to -€557,898]). There was an insignificant relative reduction of 3.1% in total emergency department use (95% CI -6.4% to 0.2%; absolute difference: -3,901, 95% CI -8,094 to 304).

### Sensitivity analyses

We conducted additional analyses to assess the robustness of our results:

1. Total attendance: Admission to hospital following an emergency department visit might indicate that the patient’s condition would not have been treatable in primary care. After excluding those patients from the sample, we continue to find no significant change in total emergency department use between comparator and intervention practices (S3 Table).
2. 6 mo intervals: The effects of the intervention were similar in January–June (immediate post intervention) and July–December (6–12 mo post intervention), but the effects were slightly larger in the latter half of the intervention period (S4 Table).
3. Regression to the mean: Our results on the main variables of interest were robust to the inclusion of baseline emergency department use (S5 Table). This suggests our main results are not reflective of random fluctuations around a long-run average.
4. Equal pre- and post-intervention comparisons: Our results on the main variables of interest were robust where only 2013 was modelled for the pre-intervention period though the magnitude of reduced minor patient-initiated attendances (total and minor attendances) is lower (higher and now significant) (S6 Table). This suggests our main results for minor patient-initiated attendances are not substantively sensitive to the pre-period analysed.
5. Model specification: Our fifth analysis concerned the modelling approach taken. We find our main variables of interest were statistically and economically robust to the use of either a negative binomial or log-linear estimation approach (S7 Table). Where differences were observed, these were for measures with rates of attendances close to zero.
6. Unmatched sample with time-trend adjustment: Our final sensitivity check estimated the effects of the intervention on the unmatched sample. Where time trends were evident, attendance was adjusted via the removal of the trend for both comparator and intervention groups. In the unmatched sample a significant difference in trends for several outcome measures in the pre-intervention period was identified between comparator and intervention groups (S8 Table). Adjusting for these, we find a smaller reduction in patient-initiated minor attendances.

## Discussion

### Principal Findings

In Greater Manchester, primary care practices that extended opening hours demonstrated a 26.4% reduction in patient-initiated referrals to the emergency department with minor problems. The pattern of results (with an impact mainly on emergency department use for minor problems) suggests that the decrease was related to the intervention. The overall impact was robust to choice of comparators and other sensitivity analyses.

The absolute decrease in emergency department use we found is lower than the number of new out-of-hours appointments booked; the ratio of additional appointments to emergency attendances avoided is approximately 3:1 (33,159 appointments booked compared to 10,933 reduced patient-initiated referrals to emergency departments).

Our findings add to a sparse literature that has investigated whether extending access to routine primary care appointments is associated with lower emergency department use. Most studies assess extended access via the introduction of out-of-hours urgent care centres or telephone triage systems and have suffered from a lack of comparator. No conclusive evidence has been found to suggest these services reduce emergency department use [8]. In general there was a noted lack of high-quality evidence to assess the associations of interventions that may reduce emergency department use. One study that is directly relevant to our study evaluated emergency department use for a group of practices with available weekend primary care appointments [32]. This study conducted a controlled before and after analysis finding similarly large reductions in emergency department attendance. Unlike our study, however, there was no control population without access to weekend appointments. The authors’ control group were those practices who did not host the appointments at weekends (but whose patients could access these services); this is more in line with addressing the question regarding how 7-day services should be delivered in primary care rather than whether introducing these services may reduce emergency department use. The authors did not consider the role of patient self-referral to emergency department use; this was a leading component in our analyses. Furthermore, the study was based on a relatively smaller population of 34 practices (four hosting the weekend appointments and 30 not hosting the appointments) covering 190,000 patients within central London, whilst our study covered a larger population of 525 practices with 2,960,354 registered patients over a wider sociodemographically diverse area.

### Strengths and Weaknesses

Our analytic design is suited to the evaluation of rapid, large scale policy interventions, such as the introduction of financial incentives or new service innovations [26,27,33,34] Nevertheless, such designs have a number of limitations compared to a formal trial, especially in (a) controlling for confounding, (b) comprehensive assessment of outcomes, and (c) standardisation of the intervention. We explore each of these issues below.

Despite efforts to control for selection outlined in the methods, it is not possible to be entirely sure that other “unmeasured” confounders were not present. The quality of the matching in the propensity score matching process is only as good as the ability to capture all differences between the intervention and comparator groups that affect treatment propensity and outcomes. Although few covariates in the propensity model were significantly different in the unmatched sample, the standardised differences (S2 Table) revealed large percentage bias. We were able to reduce the percentage bias of the model to an acceptable level of below 25%, but this is only valid for those covariates we were able to observe. The intervention and comparator groups exist within the same local environment which may mitigate the potential for unobservable macro-level differences and population differences. Should populations have changed over the period then residual confounding could be an issue. Further confounding could occur should there be differences occurring within the study period that differentially impact on emergency department use for intervention and comparator practices.

Although we explored costs, formal cost-effectiveness analysis requires detailed information on both costs and outcomes. In terms of outcomes, no data were collected on patient health outcomes. Health benefits may accrue due to extended access to “out-of-hours” primary care or via better timeliness of care; alternatively, benefits may not accrue should extended access result in substitution of appointments during routine open hours. The analysis could also not account for any additional benefits which may accrue to hospitals from reductions in emergency department use: for example, the lower concentration of use for minor problems may benefit the workflow of hospital providers. The lack of health outcomes limits an assessment of cost-effectiveness to a crude incremental cost approach based on the assumption that health benefits remain the same in both comparator and intervention practices. Taking the perspective of NHS and social care, the funding provided to the intervention practices was £3.1 million (US$4.7/€4.3 million). We find the intervention led to a cost reduction in emergency department use of £767,976 (US$1,173,890; €1,057,426), the incremental cost is therefore £2.3 million (US$3.5/€3.2 million). The intervention would therefore need to see significant health gains to be cost-effective. The evaluation was unable to disentangle set-up costs from running costs of extended hours, which may over-estimate the long-run cost of the scheme. Further research into whether the intervention is cost-effective and sustainable is required.

The implementation of the intervention was not standardised or delivered to a protocol, as in a conventional trial. The exact model of extended opening hours varied between the schemes (see Table 1), and appointment availability varied over the duration of the study. The funding provided to the schemes also supported other activities in some sites, which may have influenced outcomes. Nevertheless, there was reasonable commonality to the extension of opening hours provided in sites.

Data reporting could bias our results. There were 4,318,043 emergency department visits throughout the period (2011 quarter 2 to 2014 quarter 4). Of these, 13,792 (0.32%) had no valid practice code, and all had a valid date assigned, suggesting missing administrative data would have a negligible impact on our findings. Patients registered with a Greater Manchester practice could utilise services anywhere throughout the United Kingdom either due to movers having not registered with a new practice or due to the location of the patient at the time treatment was needed. The former is potentially problematic as the patient may not be able to use enhanced access for geographic reasons when needed. Emergency department use was measured for all patients registered with a Greater Manchester practice regardless of whether the emergency department was situated within Greater Manchester or not. Approximately 96% of emergency department visits were at departments situated within or on the border of Greater Manchester suggesting the effects of patients registered with intervention practices but not geographically able to use extended access is minimal. Finally, should reporting quality vary between hospitals, the difference-in-differences approach removes such bias provided these differences are time invariant.

### Possible Mechanisms and Explanations

Similar to conventional trials of “complex interventions,” we carried out a detailed process evaluation, which is described below.

The process evaluation explored implementation of the intervention, potential mechanisms of effect, and issues that might impact on implementation of similar schemes [35]. While the different intervention practices were comparable in their main objectives and delivery, they varied in scale and workforce arrangements (Table 1). This scope was reflected in costs of just over £1.1 million (US$1.7/€1.5 million) for the largest scheme for 2014, roughly equivalent to the three other intervention schemes combined, although it should be noted that the scale of the service meant that the extra provision was diluted to a degree, and provided the fewest appointments per 1,000 population. The scope of the scheme also raised other challenges, as the CCG struggled to engage all local practices in the area, resulting in potential inequalities of access.

The mechanisms by which access is improved are complex, depending on increased availability, affordability, and acceptability [36,37]. Extending open hours seeks to both expand availability and better align the “fit” between the system (i.e., time of day practices are open) and individuals (when patients need to access services). Although affordability is generally not an issue in the NHS, acceptability may limit the impact of improvements in availability. For example, use of professionals other than a patient’s usual primary care provider may adversely affect continuity of care. Acceptability of evening and weekend appointments among patients may also be a barrier. Our data suggest that the new services were not initially being used to capacity, and their impact may increase over time as awareness of and trust in the new service increases among patients, assuming such services can be made acceptable. The impact of extended access to out-of-hours primary care may be further improved by better “vertical” integration with hospital services.

In terms of workforce, two intervention CCGs used local staff (existing practitioner partners, salaried practitioners, and locality-based locums), while two contracted out with a local out-of-hours provider. Although care must be taken in assuming causal relationships, local staffing was associated with higher uptake of appointments. However, the workload increase for these local staff was substantial, which might threaten long-term sustainability.

### Implications

The international evidence on the effects of improved access to primary care on emergency department use is inconclusive, has rarely analysed cost data, and has lacked robust comparators [4]. Our study uses more robust methods and suggests that extending opening hours in primary care may be a useful addition to policies aiming to reduce pressures on hospital services, potentially reducing patient-initiated use of the emergency department for minor problems—but at a significant cost. Evidence on health outcomes is needed to address whether such schemes are cost-effective, this requires future evaluations to record health-related outcome data and a more comprehensive recording of costs of the intervention. The long-term effects of additional appointments and an assessment of the robustness of this study’s findings in other settings are fruitful avenues for further research. Future schemes would ideally be conducted and evaluated using more robust randomised designs [38]. Such schemes may involve greater costs of implementation, in the absence of such finance our approach provides guidance on the evaluation of future schemes where random allocation is not available.

## Supporting Information

S1 Fig Standardised percentage bias across covariates in the unmatched and matched samples. Practice characteristics obtained from the Health and Social Care Information Centre [19]. Matched sample obtained via propensity score matching using kernel matching. Deprivation measured using the Index of Multiple Deprivation, provided in tertile form by Ipsos MORI. General Practice Patient Survey sample statistics are weighted averages.
(TIF)

S1 Table Diagnostic results for alternative propensity score matching strategies. (DOCX)

S2 Table Percentage bias and variance ratios of covariates in the unmatched and matched samples. (DOCX)

S3 Table Difference-in-differences estimates from Ordinary Least Squares regressions of emergency department use: excluding admissions. (DOCX)

S4 Table Difference-in-differences estimates from Ordinary Least Squares regressions of emergency department use: January to June 2014 and July to December 2014 analysis. (DOCX)

S5 Table Difference-in-differences estimates from Ordinary Least Squares regressions of emergency department use: with baseline emergency department use. (DOCX)

S6 Table Difference-in-differences estimates from Ordinary Least Squares regressions of emergency department use: with 2013 activity for the pre-intervention period. (DOCX)

S7 Table Difference-in-differences estimates from negative binomial and Ordinary Least Squares regressions of emergency department use: comparisons of the negative binomial and log-link model specifications. (DOCX)

S8 Table Difference-in-differences estimates from Ordinary Least Squares regressions of emergency department use: with unmatched comparator group and time trend adjustment. (DOCX)

S1 Text Evaluation proposal. (PDF)

S2 Text STROBE checklist. (DOC)

## Abbreviations

CCGs: Clinical Commissioning Groups
GPs: general practitioners
NHS: National Health Service
SUS: Secondary Uses Service

## References

[1] Health & Social Care Information Centre. Hospital Episode Statistics: Accident and Emergency Attendances in England 2013–14, http://www.hscic.gov.uk/catalogue/PUB16728/acci-emer-atte-eng-2013-14-rep.pdf 2015. [Accessed 27th July 2016].

[2] Department of Health. Reference Costs 2013–14, https://www.gov.uk/government/uploads/system/uploads/attachment_data/file/380322/01_Final_2013-14_Reference_Costs_publication_v2.pdf HM Treasury 2014. [Accessed 27th July 2016].

[3] Starfield B, Shi L, Macinko J. Contribution of primary care to health systems and health. *Milbank Q* 2005;83:457–502. 16202000 10.1111/j.1468-0009.2005.00409.x PMC2690145

[4] Cowling TE, Harris MJ, Majeed A. Evidence and rhetoric about access to UK primary care. *BMJ* 2015; 350:h1513 10.1136/bmj.h1513 25828858

[5] Cowling TE, Harris MJ, Watt HC, et al Access to general practice and visits to accident and emergency (ED) departments in England: cross-sectional analysis of a national patient survey. *Br J Gen Pract* 2014; 64(624):e434–9 10.3399/bjgp14X680533 24982496 PMC4073729

[6] Monitor. Walk-in Centre Review: Final Report and Recommendations, https://www.gov.uk/government/uploads/system/uploads/attachment_data/file/283778/WalkInCentreFinalReportFeb14.pdf 2014. [Accessed 4th November 2015].

[7] Amiel C, Williams B, Ramzan F, et al Reasons for attending an urban urgent care centre with minor illness: a questionnaire study. *Emerg Med J* 2014; 10.1136/emermed-2012-202016 [published Online First: 13 January 2014]. 24421348

[8] Ismail SA, Gibbons DC, Gnani S. Reducing inappropriate accident and emergency department attendances: a systematic review of primary care service interventions. *Br J Gen Pract* 2013;63(617):e813–20 10.3399/bjgp13X675395 24351497 PMC3839390

[9] Bottle A, Tsang C, Parsons C, et al Association between patient and general practice characteristics and unplanned first-time admissions for cancer: observational study. *Br J Cancer* 2012;107:1213–9 10.1038/bjc.2012.320 [published Online First: 24 July 2012]. 22828606 PMC3494442

[10] Brettell R, Soljak M, Cecil E, et al Reducing heart failure admission rates in England 2004–2011 are not related to changes in primary care quality: national observational study. *Eur J Heart Fail* 2013;15:1335–42 10.1093/eurjhf/hft107 [published Online First: 11 July 2013]. 23845798 PMC3834843

[11] Calderon-Larranaga A, Carney L, Soljak M, et al Association of population and primary healthcare factors with hospital admission rates for chronic obstructive pulmonary disease in England: national cross-sectional study. *Thorax* 2011;66(3):191–6 10.1136/thx.2010.147058 [published Online First: 12 November 2010]. 21076143

[12] Calderon-Larranaga A, Soljak M, Cecil E, et al Does higher quality of primary healthcare reduce hospital admissions for diabetes complications? A national observational study. *Diabet Med* 2014;31:657–65 10.1111/dme.12413 [published Online First: 26 March 2014]. 24533786

[13] Calderón-Larrañaga A, Soljak M, Cowling TE, et al Association of primary care factors with hospital admissions for epilepsy in England, 2004–10: national observational study. *Seizure* 2014;23(8):657–61 24997845 10.1016/j.seizure.2014.05.008

[14] Soljak M, Calderon-Larranaga A, Sharma P, et al Does higher quality primary health care reduce stroke admissions? A national cross-sectional study. *Br J Gen Pract* 2011;61:e801–7 10.3399/bjgp11X613142 22137417 PMC3223778

[15] Imbens GW, Wooldridge JM. Recent developments in the econometrics of program evaluation. *National Bureau of Economic Research* 2008.

[16] Lechner M. The Estimation of Causal Effects by Difference-in-Difference Methods. *Foundations and Trends in Econometrics* 2010;4(3):165–224 10.1561/0800000014

[17] Meyer BD. Natural and Quasi-Experiments in Economics. *J Bus Econ Stat* 1995;13:151–161.

[18] Rosenbaum P, Rubin D. The central role of the propensity score in observational studies for causal effects. *Biometrika* 1983; (70):41–55.

[19] Health & Social Care Information Centre. General and Personal Medical Services, England– 2003–2013, As at 30 September, http://www.hscic.gov.uk/catalogue/PUB13849 2014. [Accessed 17th April 2016].

[20] Ipsos MORI. GP Patient Survey—Technical Annex 2013–2014 annual report, http://gp-survey-production.s3.amazonaws.com/archive/2014/July/1301375001_Technical%20Annex%202013-2014_FINAL%20v1.pdf Social Research Institute 2014. [Accessed 27th July 2016].

[21] Dehejia RH, Wahba S. Propensity Score Matching Methods for Nonexperimental Causal Studies. *The Review of Economics and Statistics* 2002:84(1):151–161.

[22] Heckman JJ, Ichimura H, Todd PE. Matching As An Econometric Evaluation Estimator: Evidence from Evaluating a Job Training Programme. *Review of Economic Studies* 1997:64:605–654.

[23] Sommers BD, Long SK, Baicker K. Changes in mortality after Massachusetts health care reform: A quasi-experimental study. *Annals of Internal Medicine* 2014; 60(9):585–593 10.7326/M13-2275 24798521

[24] Leuven E, Sianesi B. PSMATCH2: Stata module to perform full Mahalanobis and propensity score matching, common support graphing, and covariate imbalance testing. 2003 http://ideas.repec.org/c/boc/bocode/s432001.html. Version 4.0.11.

[25] Burbidge JB, Magee L, Robb AL. Alternative Transformations to Handle Extreme Values of the Dependent Variable. *J Am Stat Assoc* 1988;83:123–127 10.2307/2288929

[26] Harrison MJ, Dusheiko M, Sutton M et al Effect of a national primary care pay for performance scheme on emergency hospital admissions for ambulatory care sensitive conditions: controlled longitudinal study. *BMJ* 2014;349:g6423 10.1136/bmj.g6423 [published Online First: 11 November 2014]. 25389120 PMC4228282

[27] Morris S, Hunter RM, Ramsay AI et al Impact of centralising acute stroke services in English metropolitan areas on mortality and length of hospital stay: difference-in-differences analysis. *BMJ* 2014;349:g4757 10.1136/bmj.g4757 [published Online First: 5 August 2014]. 25098169 PMC4122734

[28] Austin PC. Balance diagnostics for comparing the distribution of baseline covariates between treatment groups in propensity-score matched samples. *Statistics in Medicine* 2009;28:3083–3107. 10.1002/sim.3697 19757444 PMC3472075

[29] Rubin DB. Using propensity scores to help design observational studies: Application to the tobacco litigation. *Health Services & Outcomes Research Methodology* 2001:2(3–4):169–188.

[30] Abadie A, Imbens GW. Notes and comments: Matching on the estimated propensity score. *Econometrica* 2016;84(2):781–807.

[31] Abadie A, Imbens GW. On the failure of the bootstrap for matching estimators. *Econometrica* 2008;76(6):1537–1557.

[32] Dolton P, Pathania V. Can increased primary care access reduce demand for emergency care? Evidence from England’s 7-day GP opening. *Journal of Health Economics* 2016 [In Press] 10.1016/j.jhealeco.2016.05.002 27395472

[33] Sutton M, Nikilova S, Boaden R et al Reduced mortality with hospital pay for performance in England. *New Engl J Med* 2012;367:1821–8 10.1056/NEJMsa1114951 [published Online First: 8 November 2012]. 23134382

[34] Gravelle H, Dusheiko M, Sheaff R et al Impact of case management (Evercare) on frail elderly patients: controlled before and after analysis of quantitative outcome data. *BMJ* 2007;334:31 10.1136/bmj.39020.413310.55 [published Online First: 15 November 2006]. 17107984 PMC1764106

[35] NIHR CLAHRC Greater Manchester. NHS Greater Manchester Primary Care Demonstrator Evaluation, http://clahrc-gm.nihr.ac.uk/wp-content/uploads/PCDE-final-report-full-final.pdf 2015. [Accessed 4th November 2015].

[36] Gulliford M, Figueroa-Munoz J, Morgan M et al What does 'access to health care' mean? *J Health Serv Res Policy* 2002;7(3):186–8. 12171751 10.1258/135581902760082517

[37] McIntyre D, Thiede M, Birch S. Access as a policy-relevant concept in low- and middle-income countries. *Health Econ Policy and Law* 2009;4(2):179–193 10.1017/S1744133109004836 [published Online First: 30 January 2009]. 19187569

[38] Craig P, Dieppe P, Macintyre S, Michie S, Nazareth I, Petticrew M. Developing and evaluating complex interventions: new guidance. Medical Research Council complex interventions guidance, https://www.mrc.ac.uk/documents/pdf/complex-interventions-guidance/ 2008. [Accessed 18^th^ April 2016].

