# Supplementary figures and images for: Associations between Extending Access to Primary Care and Emergency Department Visits: A Difference-In-Differences Analysis
Source: PLoS Med. 2016 Sep 6;13(9):e1002113. doi: 10.1371/journal.pmed.1002113 (PMC5012704; doi:10.1371/journal.pmed.1002113)

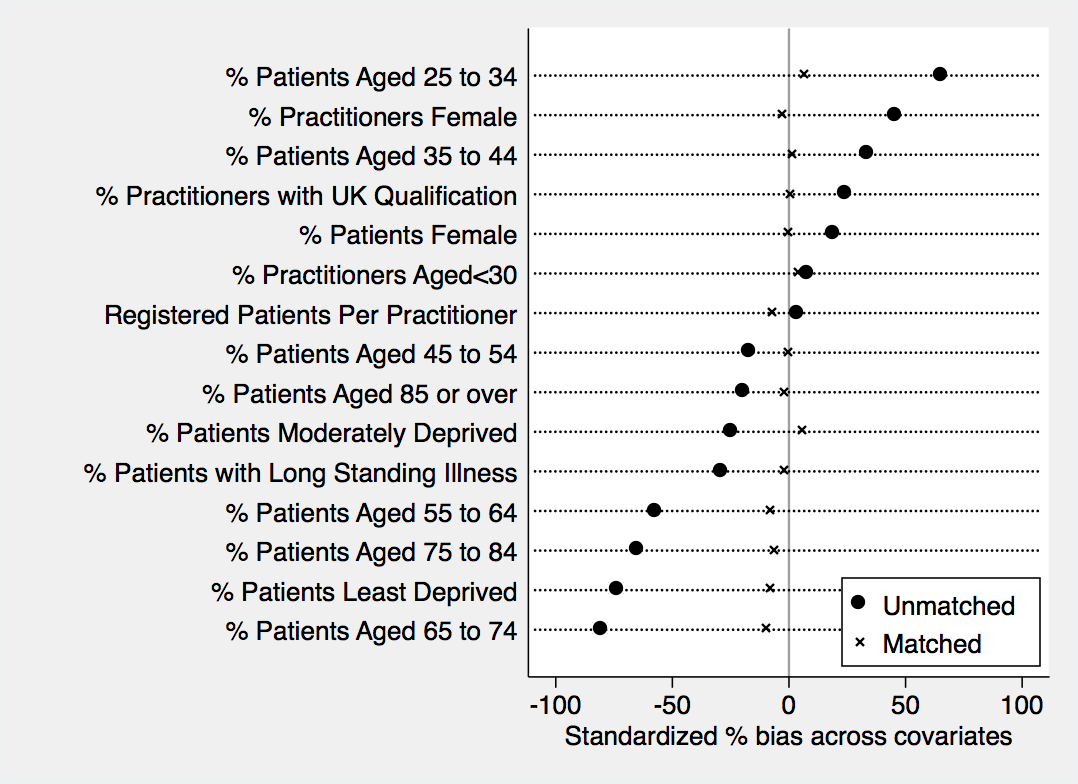

Supplement: S1 Fig — Practice characteristics obtained from the Health and Social Care Information Centre [19]. Matched sample obtained via propensity score matching using kernel matching. Deprivation measured using the Index of Multiple Deprivation, provided in tertile form by Ipsos MORI. General Practice Patient Survey sample statistics are weighted averages. (TIF) [file pmed.1002113.s001.tif]
