# Supplementary material for: Associations between Extending Access to Primary Care and Emergency Department Visits: A Difference-In-Differences Analysis
Source: PLoS Med. 2016 Sep 6;13(9):e1002113. doi: 10.1371/journal.pmed.1002113 (PMC5012704; doi:10.1371/journal.pmed.1002113)
Supplement: S1 Table — (DOCX) [file pmed.1002113.s002.docx]

| Matching procedure | Overall covariate imbalance^ | Rubin's B^^ | Rubin's R^^^ |
| --- | --- | --- | --- |
| Nearest Neighbor (=1) | 0.343 | 77.8* | 1.22 |
| Nearest Neighbor (=2) | 0.927 | 53.3* | 0.81 |
| Nearest Neighbor (=3) | 0.984 | 45.3* | 0.60 |
| Nearest Neighbor (=4) | 0.996 | 39.5* | 0.89 |
| Nearest Neighbor (=5) | 0.997 | 38.4* | 0.81 |
| Nearest Neighbor (=6) | 0.997 | 38.5* | 0.82 |
| Radius | 0.015* | 112.6* | 0.50 |
| Kernel (epanechnikov kernel) | 1.000 | 20.4 | 0.93 |
| Common support | 0.000* | 102* | 0.93 |
| Top 25% comparators & common support | 0.901 | 46.2* | 6.09* |

Caliper for all nearest neighbour matching is 0.2.

Kernel bandwidth is 0.06.

* Denotes indication of poorly matched comparator and intervention groups.

^ P-value of joint test of significance of all covariates

^^ Absolute standardised difference of the means of the linear index of the propensity score in the treated and matched non-treated group. Rubin (2001) recommends B<25[29]

^^ Ratio of treated to matched non-treated variances of the propensity score index. Rubin (2001) recommends R lies within [0.5; 2][29]
