# Supplementary material for: Associations between Extending Access to Primary Care and Emergency Department Visits: A Difference-In-Differences Analysis
Source: PLoS Med. 2016 Sep 6;13(9):e1002113. doi: 10.1371/journal.pmed.1002113 (PMC5012704; doi:10.1371/journal.pmed.1002113)
Supplement: S2 Table — (DOCX) [file pmed.1002113.s003.docx]

| Variable | Sample | Intervention | Comparator | % bias | Var(Intervention)/  Var(Comparator) |
| --- | --- | --- | --- | --- | --- |
| Practice Practitioner Characteristics |  |  |  |  |  |
| Total Number of Registered Patients per Full-time Equivalent Practitioner | U | 2049 | 2009 | 3.5 | 2.85* |
|  | M | 2049 | 2127.4 | -6.8 | 1.22 |
| % Female Practitioners | U | 0.50445 | 0.38175 | 45.9 | 0.81 |
|  | M | 0.50445 | 0.51051 | -2.3 | 0.76 |
| % Practitioners Under 30 | U | 0.01356 | 0.00939 | 8.5 | 1.15 |
|  | M | 0.01356 | 0.01122 | 4.8 | 0.94 |
| % Practitioners with UK Qualification | U | 0.71886 | 0.62886 | 24.4 | 0.75 |
|  | M | 0.71886 | 0.71389 | 1.3 | 0.93 |
| Practice Population Characteristics |  |  |  |  |  |
| % Patients Female | U | 0.51402 | 0.50226 | 19.3 | 1.45 |
|  | M | 0.51402 | 0.51399 | 0.0 | 1.11 |
| % Patients Aged 25 to 34 | U | 0.22818 | 0.17537 | 66.0 | 1.49 |
|  | M | 0.22818 | 0.22267 | 6.9 | 0.92 |
| % Patients Aged 35 to 44 | U | 0.19181 | 0.17684 | 33.6 | 1.36 |
|  | M | 0.19181 | 0.19091 | 2.0 | 1.41 |
| % Patients Aged 45 to 54 | U | 0.18083 | 0.18695 | -16.7 | 0.99 |
|  | M | 0.18083 | 0.18071 | 0.3 | 0.88 |
| % Patients Aged 55 to 64 | U | 0.13108 | 0.15523 | -57.1 | 1.52 |
|  | M | 0.13108 | 0.13438 | -7.8 | 1.26 |
| % Patients Aged 65 to 74 | U | 0.08959 | 0.12284 | -79.9 | 1.06 |
|  | M | 0.08959 | 0.09342 | -9.2 | 1.01 |
| % Patients Aged 75 to 84 | U | 0.04718 | 0.06243 | -64.3 | 1.09 |
|  | M | 0.04718 | 0.0485 | -5.6 | 0.94 |
| % Patients Aged 85 or over | U | 0.01622 | 0.02112 | -19.7 | 0.12* |
|  | M | 0.01622 | 0.01654 | -1.3 | 0.34* |
| % Patients Moderately Deprived | U | 0.22146 | 0.26153 | -24.2 | 1.22 |
|  | M | 0.22146 | 0.21193 | 5.8 | 1.12 |
| % Patients Least Deprived | U | 0.05824 | 0.18016 | -73.5 | 0.20* |
|  | M | 0.05824 | 0.07116 | -7.8 | 0.78 |
| % Patients Long Standing Illness | U | 0.48048 | 0.49856 | -28.4 | 0.75 |
|  | M | 0.48048 | 0.48177 | -2.0 | 0.61 |

U=Unmatched sample

M=Matched sample

*denotes covariates for which the variance ratio falls outside of the recommended range of [0.59; 1.71].

Practice characteristics obtained from the Health and Social Care Information Centre.[19]

Unmatched sample size is 489. There are 469 comparator practices in 2013 but data were only available for 433. Data was available for all intervention (treated) practices (56).

Matched sample obtained via propensity score matching using kernel matching. Matched sample size is 488 as 488 practices had common support for the propensity score.

Deprivation measured using the Index of Multiple Deprivation, provided in tertile form by Ipsos MORI.

General Practice Patient Survey sample statistics are weighted averages.
