# Supplementary material for: Associations between Extending Access to Primary Care and Emergency Department Visits: A Difference-In-Differences Analysis
Source: PLoS Med. 2016 Sep 6;13(9):e1002113. doi: 10.1371/journal.pmed.1002113 (PMC5012704; doi:10.1371/journal.pmed.1002113)
Supplement: S3 Table — (DOCX) [file pmed.1002113.s004.docx]

| Emergency department use | Jan-Dec 2014 | 95% confidence interval | p-value |
| --- | --- | --- | --- |
| Total (excluding admissions) | -2.70%^ | [-5.95% to 0.61%] | (0.110) |
|  |  |  |  |
| Observations | 7304 |  |  |

All activities were transformed using the inverse hyperbolic sine transformation; estimate gives the relative (risk) difference in emergency department use for intervention versus comparators; each estimate is obtained from a separate difference-in-differences Ordinary Least Squares regression.

Intervention group is matched Greater Manchester intervention practices, and comparator group is all Greater Manchester matched non-intervention practices; sample size for each model is 7,304; this is the matched (weighted) sample using kernel propensity score matching.

Bootstrapped standard errors (1,000 replications) over both propensity score and regression models.

^ Divergent time trends–the difference-in-differences assumption of equivalent time trends is not satisfied and inference should not be made on these estimates.
