# Supplementary material for: Associations between Extending Access to Primary Care and Emergency Department Visits: A Difference-In-Differences Analysis
Source: PLoS Med. 2016 Sep 6;13(9):e1002113. doi: 10.1371/journal.pmed.1002113 (PMC5012704; doi:10.1371/journal.pmed.1002113)
Supplement: S4 Table — (DOCX) [file pmed.1002113.s005.docx]

| Emergency department use | Jan-June 2014 | 95% confidence interval | p-value | July-Dec 2014 | 95% confidence interval | p-value |
| --- | --- | --- | --- | --- | --- | --- |
| Patient-initiated (minor intensity) | -23.75% | [-35.53% to -11.95%] | (<0.001) | -29.03% | [-42.52% to -15.54%] | (<0.001) |
| Cost patient-initiated (minor intensity) | -25.25% | [-37.25% to -13.25%] | (<0.001) | -28.01% | [-41.98% to -14.05%] | (<0.001) |
|  |  |  |  |  |  |  |
| Total | -1.29% | [-4.09% to 1.48%] | (0.358) | -4.88% | [-10.49% to 0.81%] | (0.093) |
| Intensity type |  |  |  |  |  |  |
| Minor | -2.28% | [-6.43% to 1.85%] | (0.278) | -6.61% | [-13.41% to 0.20%] | (0.057) |
| Standard | -4.11%^ | [-8.29% to 0.03%] | (0.052) | -6.74%^ | [-13.48% to 0.20%] | (0.057) |
| High | -2.97% | [-4.29% to 10.48%] | (0.412) | -0.81% | [-9.29% to 7.99%] | (0.883) |
| Intensity missing | 8.03% | [-2.35% to 18.41%] | (0.129) | 14.59% | [-1.80% to 27.48%] | (0.025) |
| Referral type |  |  |  |  |  |  |
| GP-referral | 4.74% | [-4.30% to 13.33%] | (0.316) | 4.11% | [-6.41% to 14.65%] | (0.443) |
| Patient-initiated | -30.39%^ | [-42.94% to -17.81%] | (<0.001) | -33.37%^ | [-47.32% to -19.34%] | (<0.001) |
| Other referral | 35.66%^ | [22.58% to 48.68%] | (<0.001) | 31.91%^ | [19.19% to 44.73%] | (<0.001) |
| Code missing | -41.51%^ | [-52.31% to -30.52%] | (<0.001) | -35.02%^ | [-45.53% to -24.46%] | (<0.001) |
|  |  |  |  |  |  |  |
| Total (excluding admissions) | -1.23%^ | [-3.93% to 1.48%] | (0.375) | -4.17%^ | [-9.83% to 1.60%] | (0.158) |
|  |  |  |  |  |  |  |
| Observations for each model | 7304 |  |  |  |  |  |

All activities were transformed using the inverse hyperbolic sine transformation; estimate gives the relative (risk) difference in emergency department use for intervention versus comparators; each estimate is obtained from a separate difference-in-differences Ordinary Least Squares regression.

Intervention group is matched Greater Manchester intervention practices, and comparator group is all Greater Manchester matched non-intervention practices; sample size for each model is 7,304; this is the matched (weighted) sample using kernel propensity score matching.

Bootstrapped standard errors (1,000 replications) over both propensity score and regression models.

^ Divergent time trends–the difference-in-differences assumption of equivalent time trends is not satisfied and inference should not be made on these estimates.
