# Supplementary material for: Associations between Extending Access to Primary Care and Emergency Department Visits: A Difference-In-Differences Analysis
Source: PLoS Med. 2016 Sep 6;13(9):e1002113. doi: 10.1371/journal.pmed.1002113 (PMC5012704; doi:10.1371/journal.pmed.1002113)
Supplement: S5 Table — (DOCX) [file pmed.1002113.s006.docx]

| Emergency department use | Jan-Dec 2014 | 95% confidence interval | p-value |
| --- | --- | --- | --- |
| Patient-initiated referrals (minor intensity) | -26.41% | [-38.40% to -14.36%] | (<0.001) |
| Cost of patient-initiated referrals (minor intensity) | -26.65% | [-39.00% to -14.26%] | (<0.001) |
|  |  |  |  |
| Total | -3.10% | [-6.36% to 0.22%] | (0.067) |
| Intensity type |  |  |  |
| Minor | -4.46% | [-9.09% to 0.19%] | (0.060) |
| Standard | -5.44% | [-9.84% to 0.92%] | (0.018) |
| High | -1.00% | [-5.57% to 8.01%] | (0.724) |
| Intensity missing | 11.29% | [0.84% to 21.83%] | (0.034) |
| Referral type |  |  |  |
| GP-referral | 4.49% | [-4.04% to 12.68%] | (0.312) |
| Patient-initiated referrals | -31.91%^ | [-44.62% to -19.09%] | (<0.001) |
| Other referral | 33.81%^ | [21.51% to 46.08%] | (<0.001) |
| Code missing | -38.27%^ | [-48.49% to -27.92%] | (<0.001) |
|  |  |  |  |
| Observations for each model | 7304 |  |  |

All activities were transformed using the inverse hyperbolic sine transformation; estimate gives the relative (risk) difference in emergency department use for intervention versus comparators; each estimate is obtained from a separate difference-in-differences Ordinary Least Squares regression.

Intervention group is matched Greater Manchester intervention practices, and comparator group is all Greater Manchester matched non-intervention practices; sample size for each model is 7,304; this is the matched (weighted) sample using kernel propensity score matching.

Bootstrapped standard errors (1,000 replications) over both propensity score and regression models.

^ Divergent time trends–the difference-in-differences assumption of equivalent time trends is not satisfied and inference should not be made on these estimates.
