# Supplementary material for: Associations between Extending Access to Primary Care and Emergency Department Visits: A Difference-In-Differences Analysis
Source: PLoS Med. 2016 Sep 6;13(9):e1002113. doi: 10.1371/journal.pmed.1002113 (PMC5012704; doi:10.1371/journal.pmed.1002113)
Supplement: S6 Table — (DOCX) [file pmed.1002113.s007.docx]

| Emergency department use | Jan-Dec 2014 | 95% confidence interval | p-value |
| --- | --- | --- | --- |
| Patient-initiated referrals (minor intensity) | -22.27% | [-32.19% to -12.19%] | (<0.001) |
| Cost of patient-initiated referrals (minor intensity) | -21.42% | [-31.74% to -10.94%] | (<0.001) |
|  |  |  |  |
| Total | -3.79% | [-6.88% to -0.62%] | (0.019) |
| Intensity type |  |  |  |
| Minor | -5.18% | [-8.89% to -1.37%] | (0.008) |
| Standard | -2.82% | [-6.96% to 1.40%] | (0.192) |
| High | -2.82% | [-9.15% to 3.82%] | (0.420) |
| Intensity missing | 2.09% | [-4.12% to 8.29%] | (0.510) |
| Referral type |  |  |  |
| GP-referral | 4.10% | [-3.68% to 11.95%] | (0.300) |
| Patient-initiated referrals | -23.72% | [-34.44% to -12.85%] | (<0.001) |
| Other referral | 22.70% | [12.19% to 33.32%] | (<0.001) |
| Code missing | -0.18% | [-3.95% to 3.68%] | (0.939) |
|  |  |  |  |
| Observations for each model | 3888 |  |  |

All activities were transformed using the inverse hyperbolic sine transformation; estimate gives the relative (risk) difference in emergency department use for intervention versus comparators; each estimate is obtained from a separate difference-in-differences Ordinary Least Squares regression.

Intervention group is matched Greater Manchester intervention practices, and comparator group is all Greater Manchester matched non-intervention practices; sample size for each model is 3,888; this is the matched (weighted) sample using kernel propensity score matching.

Bootstrapped standard errors (1,000 replications) over both propensity score and regression models.
