# Supplementary material for: Associations between Extending Access to Primary Care and Emergency Department Visits: A Difference-In-Differences Analysis
Source: PLoS Med. 2016 Sep 6;13(9):e1002113. doi: 10.1371/journal.pmed.1002113 (PMC5012704; doi:10.1371/journal.pmed.1002113)
Supplement: S7 Table — (DOCX) [file pmed.1002113.s008.docx]

|  | Negative binomial |  |  | Log-linear |  |  | Difference |
| --- | --- | --- | --- | --- | --- | --- | --- |
| Emergency department use | Jan-Dec 2014 | 95% confidence interval | p-value | Jan-Dec 2014 | 95% confidence interval | p-value |  |
| Patient-initiated referrals (minor intensity) | -25.08% | [-35.34% to -14.82%] | (<0.001) | -26.39% | [-36.65% to -16.13%] | (<0.001) | 1.31pp |
| Cost of patient-initiated referrals (minor intensity) | -26.14% | [-36.96% to -15.32%] | (<0.001) | -26.63% | [-37.11% to -16.16%] | (<0.001) | 0.49pp |
|  |  |  |  |  |  |  |  |
| Total | -2.29% | [-4.88% to -0.29%] | (0.082) | -3.08% | [-5.98% to -0.19%] | (0.037) | 0.79pp |
| Intensity type |  |  |  |  |  |  |  |
| Minor | -3.65% | [-7.45% to 0.15%] | (0.060) | -4.45% | [-8.57% to -0.32%] | (0.035) | 0.80pp |
| Standard | -4.85% | [-8.51% to -1.18%] | (0.010) | -5.42% | [-9.43% to -1.41%] | (0.008) | 0.57pp |
| High | 3.16% | [-1.92% to 8.24%] | (0.222) | 1.08% | [-4.45% to 6.61%] | (0.701) | 2.08pp |
| Intensity missing | 31.57% | [-6.77% to 69.90%] | (0.107) | 11.31% | [2.40% to 20.22%] | (0.013) | 20.26pp |
| Referral type |  |  |  |  |  |  |  |
| GP-referral | 8.92% | [-0.59% to 18.43%] | (0.066) | 4.43% | [-3.45% to 12.30%] | (0.270) | 4.49pp |
| Patient-initiated referrals | -30.17% | [-41.25% to -19.09%] | (<0.001) | -31.88% | [-42.80% to -20.96%] | (<0.001) | 1.71pp |
| Other referral | 27.18% | [19.87% to 34.49%] | (<0.001) | 33.79% | [23.36% to 44.22%] | (<0.001) | -6.61pp |
| Code missing | -78.92% | [-109.50% to -48.34%] | (<0.001) | -38.27% | [-49.32% to -27.21%] | (<0.001) | -40.65pp |
|  |  |  |  |  |  |  |  |
| Observations for each model | 7304 |  |  | 7304 |  |  |  |

pp: percentage point

All activities were transformed using the inverse hyperbolic sine transformation; estimate gives the relative (risk) difference in emergency department use for intervention versus comparators; each estimate is obtained from a separate difference-in-differences regression.

Intervention group is matched Greater Manchester intervention practices, and comparator group is all Greater Manchester matched non-intervention practices; sample size for each model is 7,304; this is the matched (weighted) sample using kernel propensity score matching.
