# Supplementary material for: Associations between Extending Access to Primary Care and Emergency Department Visits: A Difference-In-Differences Analysis
Source: PLoS Med. 2016 Sep 6;13(9):e1002113. doi: 10.1371/journal.pmed.1002113 (PMC5012704; doi:10.1371/journal.pmed.1002113)
Supplement: S8 Table — (DOCX) [file pmed.1002113.s009.docx]

| Emergency department use | Jan-Dec 2014 | 95% confidence interval | p-value |
| --- | --- | --- | --- |
| Patient-initiated referrals (minor intensity) | -18.81%^ | [-25.06% to -12.56%] | (<0.001) |
| Cost of patient-initiated referrals (minor intensity) | -16.04%^ | [-22.50% to -9.58%] | (<0.001) |
|  |  |  |  |
| Total | -2.66% | [-5.32% to -0.002%] | (0.050) |
| Intensity type |  |  |  |
| Minor | -8.35%^ | [-11.80% to -4.90%] | (<0.001) |
| Standard | -0.34%^ | [-3.40% to 2.72%] | (0.828) |
| High | -2.31% | [-6.91% to 2.29%] | (0.324) |
| Intensity missing | 7.97% | [0.50% to 15.45%] | (0.037) |
| Referral type |  |  |  |
| GP-referral | 4.37% | [-2.59% to 11.33%] | (0.218) |
| Patient-initiated referrals | -10.85%^ | [-17.47% to -4.24%] | (0.001) |
| Other referral | 4.26%^ | [-3.10% to 11.62%] | (0.256) |
| Code missing | 67.91%^ | [57.25% to 78.58%] | (<0.001) |
|  |  |  |  |
| Observations for each model | 7433 |  |  |

All activities were transformed using the inverse hyperbolic sine transformation; estimate gives the relative (risk) difference in emergency department use for intervention versus comparators; each estimate is obtained from a separate difference-in-differences Ordinary Least Squares regression.

Intervention group is all Greater Manchester intervention practices, and comparator group is all Greater Manchester non-intervention practices; sample size for each model is 7,433.

^ Divergent time trends–the estimate provided is in relation to time-trend adjusted attendance.
