# Supplementary material for: Associations between Extending Access to Primary Care and Emergency Department Visits: A Difference-In-Differences Analysis
Source: PLoS Med. 2016 Sep 6;13(9):e1002113. doi: 10.1371/journal.pmed.1002113 (PMC5012704; doi:10.1371/journal.pmed.1002113)
Supplement: S1 Text — (PDF) [file pmed.1002113.s010.pdf]

CLAHRC GM Demonstrator Pilot Evaluation: Proposal

Six primary care demonstrator communities have been established by NHS Greater Manchester, with the aim of testing various elements of the newly developed *Primary Care Commissioning Strategy for Greater Manchester*. While the pilot communities differ in their scope, size and ambitions, it is possible to identify three core themes common to all: **integration**, **access** and **technology**. These themes form the main basis of this evaluation and are consistent with those found within current national and regional policies concerning the desired movement of services and activity away from secondary care services and into primary care, or, from a patient's perspective, 'moving care closer to home'.

The key questions the evaluation aims to answer in relation to the demonstrator communities, and the specific interventions they are piloting, are: **What appears to work? What doesn't appear to work?** and, in both cases, crucially, **Why?** Importantly, the evaluation is designed in such a way that ensures that knowledge and learning gathered over the course of the evaluation can be shared with, and across, the pilot sites in real-time, enabling them to adapt and refine their interventions 'as they go' in order to maximise their potential impact. More details about the evaluation approach adopted here can be found in a separate briefing (suitable for wider circulation): "CLAHRC GM Demonstrator Pilot Evaluation: An overview and rationale"

The evaluation consists of two linked elements: a **process evaluation** and an **outcome evaluation.**

The process evaluation

The **process evaluation** will aim to explore the process by which pilot interventions consistent with the key themes of integration, access and technology, are being defined, implemented and modified over time within each demonstrator site.

The first step in this process will involve the gathering of qualitative data from a range of stakeholders within each of the demonstrator communities, to gain an in-depth and detailed understanding of the interventions themselves, what they are intended to achieve and how their impact is to be measured by the pilot site. Across the pilots, these initial questions will enable us to identify:

- How integration/access/technology is being defined by each pilot.
- What specific service interventions are being used by each pilot to address the key issues of integration/access/use of technology.
- How improvements associated with integration/access/use of technology are being measured by each pilot.

Using this as a foundation, the process evaluation will then explore how the process of implementing these interventions unfolds in reality. This will be fundamental in enabling us to understand what appears

to work, what doesn't appear to work, and in both cases, why? Particular attention will be paid, at this stage, to the impact the local context has on how the interventions being deployed are being implemented (e.g. what helps or hinders their implementation) and how this impacts on their known, or perceived, effectiveness.

Already, in the early stages of the evaluation (March 2014), a number of factors have been identified which appear to affect how the various interventions are implemented. These include:

- Information technology and governance (e.g. sharing records, integration of IT system, processes of consent, governance of web-based confidential content).
- Workforce issues (e.g. staff skill mix, developmental needs, satisfaction, contracting).
- Organisational development (e.g. establishment of provider federations, engagement and partnership management across sector boundaries, resistance to new ways of working).
- Finance (e.g. cost of innovations, sustainability of costs, feasibility of scaling and spreading innovation).

Information gathered over the course of the process evaluation will not only be used to evaluate and inform the development of interventions within the demonstrator communities themselves, but will also be used to identify whether, and how, the interventions being tested could be scaled up and applied across a larger population. This will be a fundamental measure of success for each pilot, examining to what extent, if any, the piloted interventions are sustainable and scalable in the longer term and therefore the extent to which they offer solutions to the endemic problems associated with integration and access in primary care.

What data will the process evaluation use?

Data for the process evaluation will be principally by gathered through semi-structured, in-depth interviews with key personnel and stakeholders in each of the demonstrator communities. This will be supported and supplemented by members of the evaluation team attending and participating in relevant meetings, clinical forums and learning events, direct observations of pilot site activities (where possible) and analysis of key documents related to the activities taking place in demonstrator (e.g. meeting agendas and minutes, proposals, plans and protocols).

The outcome evaluation

The **outcome evaluation** will aim to help establish, as far as possible, the effectiveness of the interventions being implemented within each pilot site. While it is unlikely that this data will be able to definitively prove whether an intervention has or has not achieved its desired outcome, for reasons documented elsewhere (see breifing "CLAHRC GM Demonstrator Pilot Evaluation: An overview and rationale), the outcome evaluation will, together with the process data, provide an *indication* as to whether an intervention has or has not worked.

What data will the outcome evaluation use?

The outcome evaluation will use a range of routinely collected data to monitor healthcare utilization at baseline and again 6 and 12 months after the start of the demonstrator pilots. This will include data related to the use of both primary and secondary care services, obtained via the Data Linkage and Extract Service provided by the Health and Social Care Information Centre (subject to all necessary approvals).

The General Practice Extraction Service will be used to link primary and secondary care data to enable monitoring. Two sets of data will form the main basis of the outcome evaluation:

1. A&E attendances, emergency admissions, emergency readmissions and resource use

We will assess impact on A&E attendance, emergency admissions, readmissions within 30 days, and resource use at inpatient stays measured using HRG tariffs and the length of stay of hospital admissions. This requires bespoke extracts of Hospital Episode Statistics (HES) from the Data Linkage and Extract Service at the Health and Social Care Information Centre. We will request patient level data which will enable us to aggregate to appropriate levels (e.g. CCG, acute NHS Trust and/or practice level) to link the analysis of the impact of the pilot interventions to the population of interest.

HES data will initially be extracted for an 18-month period from 01/10/12 to 31/03/14 to facilitate analysis of the pilots both before and during the intervention phase. There is a 6-month lag between collection and release of HES data but where feasible additional analyses can be undertaken for 6-months after the intervention phase to isolate whether any identified changes in A&E activity persist.

2. Patient reported access to and satisfaction with GP services

We will use data from the General Practice Patient Survey (GPPS) to assess improvements in patient reported access to and satisfaction with GP services (e.g. out of hours, extended hours in general practice). The GPSS is completed on average by 200 patients per practice and includes core items relating to access to services and satisfaction with providers and care. Only summary data is published on publicly accessible web spaces but we will negotiate with NHS England to gain access to full data for all items included in this survey.

Results are published every June and December, and include data from the two latest waves.

Data collection and analysis schedule

The evaluation will be conducted over several linked phases, as follows:

Phase	Data collection and analysis activities
Phase One<br>January-March 2014	Interviews with pilot leads, with the primary purpose of:<br><br>• Describing the context and objectives of each pilot<br>• Establishing parameters for qualitative and quantitative analysis<br>• Identifying key personnel who will participate in further data collection<br><br>Interview with regional NHS England lead, with the primary purpose of:<br><br>• Situating the pilots in the context of the broader Primary Care Commissioning Strategy<br>• Describing the process according to which demonstrator sites were selected<br>• Identifying the longer term objectives for these, and other sites, beyond the initial pilot phase

Phase Two<br>March-May 2014	Interviews and observations in each pilot area, in collaboration with key personnel identified in Phase One, to enable the implementation process to be examined and evaluated in real-time.<br><br>• Specific interventions will be selected from each site on the basis of their alignment with the core themes- integration, access and technology.<br>• The implementation process for each intervention will be studied, taking in a range of stakeholder perspectives<br>• Analysis will be developed of the organisational processes that support or impede successful implementation of each intervention<br>• Rapid reviews will be conducted in order to collate and feedback research evidence on areas relevant to each pilot
Phase Three<br>May-October 2014	Comparison of the implementation process between and across sites for each of the three themes: integration, access and technology<br><br>Provision of formal feedback to each site, providing site-specific information and learning, as well as learning from across all of the demonstrator communities.<br><br>Triangulation of qualitative and quantitative data analyses:<br><br>• Comparison of baseline quantitative data and that collected at 6 and 12 months, to evaluate and identify changes in healthcare utlisation<br>• Analysis of qualitative and quantitative analyses for points of convergence and divergence, to develop implementation strategies which will guide service design and implementation beyond the pilot phase and initial pilot sites

Outputs

It is intended that the findings of the evaluation will be shared with, and across, pilot sites in 'real-time' to enable pilot interventions to be modified, adapted and improved on an ongoing basis.

The main output will be a written report (approx. 10,000) words delivered in December 2014 to NHS England (Greater Manchester) and the pilots themselves. Opportunities will then be provided for demonstrator sites to provide comment and feedback, which will be incorporated into a final report to be submitted in March 2015.

We will provide interim summaries of findings as required by stakeholders throughout Greater Manchester, usually in the form of presentations of key data or emergent issues.

The cyclical approach to research and practice adopted in this evaluation involves a continuous process of feedback and learning between research and practice. In addition, formal feedback will be organised: at least two feedback sessions per pilot site, and at least two shared learning events involving all sites together.

Additionally there will be participation in relevant local and regional health forums, presentations at regional, national and international conferences, and publications in both practitioner oriented and academic journals.

Personnel

The evaluation is being carried out by a team from the NIHR (National Institute for Health Research) Collaboration for Leadership in Applied Health Research and Care (CLAHRC) Greater Manchester. CLAHRC Greater Manchester is a partnership between the National Institute for Health Research (NIHR) providers and commissioners from the NHS, industry, the third sector and the University of Manchester. The demonstrator evaluation is part of the programme of work in the Primary Care theme of CLAHRC GM with involvement from a range of academic and non-academic partners:

Dr Damian Hodgson (Evaluation Lead), Manchester Business School, University of Manchester
Dr Simon Bailey (Process Evaluation Lead), Manchester Business School, University of Manchester
Dr Peter Coventry (Outcome Evaluation Lead), Centre for Primary Care, University of Manchester
Ms Katy Rothwell, Salford Royal NHS Foundation Trust
Dr Rebecca Elvey, Centre for Primary Care, University of Manchester
Prof Kath Checkland, Centre for Primary Care, University of Manchester
Prof Peter Bower, Centre for Primary Care, University of Manchester
Dr Christian Blickem, Centre for Primary Care, University of Manchester
Dr Stephen Parkin, Centre for Primary Care, University of Manchester
Dr Søren Rud Kristensen, Centre for Health Economics, University of Manchester
Dr William Whittaker, Centre for Health Economics, University of Manchester
Prof Matt Sutton, Centre for Health Economics, University of Manchester
Prof Ruth Boaden, Manchester Business School, University of Manchester

Please direct all enquires to: simon.bailey@mbs.ac.uk
