# Supplementary material for: Associations between Extending Access to Primary Care and Emergency Department Visits: A Difference-In-Differences Analysis
Source: PLoS Med. 2016 Sep 6;13(9):e1002113. doi: 10.1371/journal.pmed.1002113 (PMC5012704; doi:10.1371/journal.pmed.1002113)
Supplement: S2 Text — (DOC) [file pmed.1002113.s011.doc]

STROBE Statement—Checklist of items that should be included in reports of ***case-control studies***

|  | | Item No | Recommendation | Article reference |
| --- | --- | --- | --- | --- |
| **Title and abstract** | | 1 | (*a*) Indicate the study’s design with a commonly used term in the title or the abstract | Title page and abstract |
|  |  |  | (*b*) Provide in the abstract an informative and balanced summary of what was done and what was found | Abstract |
| Introduction | | | |  |
| Background/rationale | | 2 | Explain the scientific background and rationale for the investigation being reported | Introduction: Paragraphs 1 to 4 |
| Objectives | | 3 | State specific objectives, including any prespecified hypotheses | Introduction:  Paragraph 5 |
| Methods | | | |  |
| Study design | | 4 | Present key elements of study design early in the paper | Abstract  Methods:  Paragraphs 6 to 12 |
| Setting | | 5 | Describe the setting, locations, and relevant dates, including periods of recruitment, exposure, follow-up, and data collection | Methods:  Paragraphs 2 to 5; 8 to 9  Tables 1 and 2, Figures 1 to 3 |
| Participants | | 6 | (*a*) Give the eligibility criteria, and the sources and methods of case ascertainment and control selection. Give the rationale for the choice of cases and controls | Methods:  Paragraph 9 |
|  |  |  | (*b*) For matched studies, give matching criteria and the number of controls per case | Methods:  Paragraphs 15 to 17 |
| Variables | | 7 | Clearly define all outcomes, exposures, predictors, potential confounders, and effect modifiers. Give diagnostic criteria, if applicable | Methods:  Outcomes - Paragraphs 6 to 7; 20  Exposures – Paragraphs 8; 18 to 19; Table 1  Predictors – Paragraphs 10 to 12; 20  Potential confounders and effect modifiers  – Paragraphs 13 to 19  Diagnostic criteria - Box 1 |
| Data sources/ measurement | | 8* | For each variable of interest, give sources of data and details of methods of assessment (measurement). Describe comparability of assessment methods if there is more than one group | Methods:  Paragraphs 6 to 9; 12 to 19  Diagnostic criteria - Box 1  Table S1 and S2 |
| Bias | | 9 | Describe any efforts to address potential sources of bias | Methods:  Paragraphs 13 to 21 |
| Study size | | 10 | Explain how the study size was arrived at | Methods:  Paragraph 9 |
| Quantitative variables | | 11 | Explain how quantitative variables were handled in the analyses. If applicable, describe which groupings were chosen and why | Methods:  Paragraphs 6 to 12; 14 to 17; 18 to 21  Diagnostic criteria - Box 1 |
| Statistical methods | | 12 | (*a*) Describe all statistical methods, including those used to control for confounding | Methods:  Paragraphs 10 to 21 |
|  |  |  | (*b*) Describe any methods used to examine subgroups and interactions | Methods:  Paragraph 21 |
|  |  |  | (*c*) Explain how missing data were addressed | Methods:  Paragraphs 6; 9 |
|  |  |  | (*d*) If applicable, explain how matching of cases and controls was addressed | Methods: Paragraph 13 to 17; 21 |
|  |  |  | (*e*) Describe any sensitivity analyses | Methods:  Paragraph 21 |
| Results | | | |  |
| Participants | | 13* | (a) Report numbers of individuals at each stage of study—eg numbers potentially eligible, examined for eligibility, confirmed eligible, included in the study, completing follow-up, and analysed | Methods:  Paragraph 9 |
|  |  |  | (b) Give reasons for non-participation at each stage | N/A see Discussion: Paragraph 7 |
|  |  |  | (c) Consider use of a flow diagram | N/A |
| Descriptive data | | 14* | (a) Give characteristics of study participants (eg demographic, clinical, social) and information on exposures and potential confounders | Results:  Paragraphs 1 to 3  Tables S1 and S2  Figures 1 to 3  Figure S1 |
|  |  |  | (b) Indicate number of participants with missing data for each variable of interest | Discussion: paragraph 7 |
| Outcome data | | 15* | Report numbers in each exposure category, or summary measures of exposure | Results:  Paragraph 1  Figure 2 |
| Main results | | 16 | (*a*) Give unadjusted estimates and, if applicable, confounder-adjusted estimates and their precision (eg, 95% confidence interval). Make clear which confounders were adjusted for and why they were included | Results:  Paragraphs 5 to 7  Tables 2; S1; S2; S3; S4; S5; S6; S7; S8  Figure S1 |
|  |  |  | (*b*) Report category boundaries when continuous variables were categorized | N/A |
|  |  |  | (*c*) If relevant, consider translating estimates of relative risk into absolute risk for a meaningful time period | Results:  Paragraph 6 |
| Other analyses | 17 | Report other analyses done—eg analyses of subgroups and interactions, and sensitivity analyses | | Results:  Paragraph 7  Tables S1; S2; S3; S4; S5; S6; S7; S8 |
| Discussion | | | |  |
| Key results | 18 | Summarise key results with reference to study objectives | | Discussion:  Paragraph 1 to 3; 13 |
| Limitations | 19 | Discuss limitations of the study, taking into account sources of potential bias or imprecision. Discuss both direction and magnitude of any potential bias | | Discussion:  Paragraphs 4 to 8; 13 |
| Interpretation | 20 | Give a cautious overall interpretation of results considering objectives, limitations, multiplicity of analyses, results from similar studies, and other relevant evidence | | Discussion:  Paragraphs 3 to 13 |
| Generalisability | 21 | Discuss the generalisability (external validity) of the study results | | Discussion:  Paragraphs 4 to 13 |
| Other information | | | |  |
| Funding | 22 | Give the source of funding and the role of the funders for the present study and, if applicable, for the original study on which the present article is based | | Additional Information |

*Give information separately for cases and controls.

**Note:** An Explanation and Elaboration article discusses each checklist item and gives methodological background and published examples of transparent reporting. The STROBE checklist is best used in conjunction with this article (freely available on the Web sites of PLoS Medicine at http://www.plosmedicine.org/, Annals of Internal Medicine at http://www.annals.org/, and Epidemiology at http://www.epidem.com/). Information on the STROBE Initiative is available at http://www.strobe-statement.org.
